# Dietary Intake of Carotenoid-Rich Vegetables Reduces Visceral Adiposity in Obese Japanese men—A Randomized, Double-Blind Trial

**DOI:** 10.3390/nu12082342

**Published:** 2020-08-05

**Authors:** Tomohisa Takagi, Ryotaro Hayashi, Yuji Nakai, Shinji Okada, Rumiko Miyashita, Mayumi Yamada, Yoichi Mihara, Katsura Mizushima, Mayuko Morita, Kazuhiko Uchiyama, Yuji Naito, Yoshito Itoh

**Affiliations:** 1 Molecular Gastroenterology and Hepatology, Graduate School of Medical Science, Kyoto Prefectural University of Medicine, Kyoto 602-8566, Japan; mizusima@koto.kpu-m.ac.jp (K.M.); k-uchi@koto.kpu-m.ac.jp (K.U.); ynaito@koto.kpu-m.ac.jp (Y.N.); yitoh@koto.kpu-m.ac.jp (Y.I.); 2 Department for Medical Innovation and Translational Medical Science, Graduate School of Medical Science, Kyoto Prefectural University of Medicine, Kyoto 602-8566, Japan; 3 Nippon Flour Mills Co., Ltd., Innovation Center, Kanagawa 243-0041, Japan; r-hayashi@nippn.co.jp (R.H.); miyashita@nippn.co.jp (R.M.); 4 Section of Food Sciences, Institute of Regional Innovation, Hirosaki University, Aomori 038-0012, Japan; yunakai@hirosaki-u.ac.jp; 5 Graduate School of Agricultural and Life Sciences, The University of Tokyo, Tokyo 113-8654, Japan; asoka@mail.ecc.u-tokyo.ac.jp; 6 Clinical Research Support Office, National Cancer Center Hospital East, Kashiwa 277-8577, Japan; mayyamad@east.ncc.go.jp; 7 NK Medico co., LTD., Tokyo 105-0012, Japan; y.mihara@noritsu-koki.com; 8 Health Care Nutrition, Showa Gakuin Junior College, Chiba 272-0823, Japan; m-morita@koto.kpu-m.ac.jp

**Keywords:** carotenoids, lycopene, lutein, visceral adiposity, metabolic syndrome

## Abstract

Metabolic syndrome, whose main diagnostic component is obesity, is a risk factor for lifestyle-related diseases, type 2 diabetes, and cardiovascular disease. Diet is known to affect the prevalence of metabolic syndrome. However, the effect of diet on metabolic syndrome in Japanese subjects has not been thoroughly explored. In the present study, we investigated the effect of carotenoid-rich vegetables, particularly lycopene- and lutein-rich vegetables, on the metabolic syndrome in obese Japanese men. We conducted an 8-week long randomized, double-blinded, controlled clinical trial in which, 28 middle-aged (40 ≤ age < 65) Japanese men with high body mass index (BMI ≥ 25) were randomized into four dietary groups: high lycopene + high lutein (HLyHLu), high lycopene + low lutein (HLyLLu), low lycopene + high lutein (LLyHLu), and low lycopene + low lutein (LLyLLu). Our results showed that daily beverage-intake increased the plasma levels of carotenoids without adverse effects, and the visceral fat level was significantly decreased in all the groups. The waist circumference was significantly decreased only in the HLyLLu group, whereas the CoQ10 oxidation rate was decreased in all the groups. The gene expression profiles of whole blood samples before and after ingestion differed only in the LLyLLu group, indicating the effect of carotenoids on gene expression profile. In conclusion, our results suggest that dietary uptake of carotenoid-rich vegetables increases their concentration in blood and reduces the intra-abdominal visceral fat.

## 1. Introduction

One of the main diagnostic components of metabolic syndrome is obesity, which is usually measured by the waist circumference and the intra-abdominal visceral fat, in addition to dyslipidemia (the condition of raised triglycerides and reduced high density lipoprotein (HDL)-cholesterol in blood); other components are raised blood pressure and fasting plasma glucose, all of which are related to weight gain. In Japan, the prevalence of obesity (body mass index (BMI) ≥ 25) has been reported to be 30.4% in males and 21.1% in females [1], and the metabolic syndrome has been linked to an increased risk of type 2 diabetes and cardiovascular disease [2]; thus, the promotion of the appropriate body weight management has been an important issue for the national health program.

Both positive and negative relationships between metabolic syndrome and diet have been reported; on the one hand, it was shown that the Mediterranean diet reduced the prevalence of metabolic syndrome and the associated cardiovascular risk by reducing the systemic vascular inflammation and endothelial dysfunction [3]. On the other hand, western diet raises the risk of metabolic syndrome. Thus, diet affects the prevalence of metabolic syndrome [4].

Carotenoids are yellow or red lipid-soluble pigments, and they are widely distributed in nature. Many of the carotenoid pigments are synthesized by photosynthetic organisms, including plants and microorganisms. Thus far, over 750 naturally occurring carotenoids have been reported [5]. Thirty-four types of carotenoids and their metabolites have been detected in human plasma [6]. In mammals, β-carotene, α-carotene, and β-cryptoxanthin are converted to retinal by cleavage dioxygenases and represent the main dietary sources of vitamin A [7]. However, lycopene and lutein were not reported to possess pro-vitamin A activity and/or biological antioxidant activity [8,9,10]. Therefore, in this study we focused on the effects of lycopene and lutein on the visceral adipose tissue of obese Japanese men.

Intake of carotenoids has been associated with risk reduction of lifestyle-related diseases [11,12,13,14] and high lycopene levels on plasma have been associated with a reduction of the cardiovascular disease risk [15]. Some reports described potential mechanisms of lycopene function for the prevention of lifestyle-related diseases. For instance, a lycopene-containing tomato product was proven capable of reducing the risk of arteriosclerosis and ischemic heart disease by repressing the generation of serum fatty acid peroxides [16]. Lycopene prevented cardiovascular disease by decreasing the level of low density lipoprotein (LDL) synthesis through reduction of the 3-hydroxy-3-methylglutaryl coenzyme A (HMG-CoA) [17]. Moreover, a separate study indicated that lycopene could prevent cardiovascular disease through improvement of the HDL functionality by stimulating the activity of the lecithin cholesterol acyltransferase (LCAT) [18].

Lutein was connected with the improvement of cognitive function and the proposed mechanism suggested that an increase in central lutein improved the neuronal efficiency and enhanced the neural conduction [19]. Lutein also increased the brain-derived neurotrophic factor (BDNF) [20], and it was suggested that lutein could possibly maintain the brain function by decreasing the oxidative stress [21]. Moreover, it was reported that lutein supplementation improved the macular pigment optical density both in patients with age-related macular degeneration and in healthy subjects [22]. It has been suggested that StARD3, a human retinal lutein-binding protein expressed in macula, promoted the accumulation of lutein and the improvement of the macular pigment optical density [23]. Data on the correlation between lutein and metabolic syndrome are conflicting. A higher dietary intake and higher blood concentrations of lutein are generally associated with better cardiometabolic health [24], whereas it has been demonstrated that serum level of lutein was not related to body fat [25].

Furthermore, many kinds of carotenoids are included in various vegetables and fruits, and increased consumption of such food has been related with reduced risk for cardiovascular disease, diabetes, and cancer [26,27,28]. Thus, consumers are beginning to be appreciative of the potential benefit that the active diet of vegetables and fruits could bring regarding disease prevention through the maintenance and improvement of their overall health [29]. However, the daily intake of vegetables and fruits for the Japanese adult is less than the globally recognized daily required quantities of 350 and 200 g of vegetables and fruits, respectively.

Thus far, there have been only limited clinical intervention studies on vegetables regarding their effects on lifestyle-related diseases, including obesity. Therefore, in the present study, we conducted the trial in order to evaluate the functionality of various carotenoid-rich vegetables to the Japanese human health focusing on visceral adiposity in obese. In particular, we focused on the effects of lycopene and lutein and we investigated how they contribute to it.

## 2. Materials and Methods

### 2.1. Study Design

This study was a randomized, double-blind controlled (RCT) study, 8 weeks in duration from 14 May 2014 to 11 June 2014. The study protocol was approved by the Ethics Committee of Kyoto Prefectural University of Medicine (approved number: ERB-C-111), and all subjects provided informed consent. The eligibility of each subject was evaluated based on full review of their clinical history and physical examination, as well as on full blood count and serum chemistry. Selected eligible subjects were of middle age (40 ≤ age < 65), with high BMI (BMI ≥ 25). Subjects who had participated in other clinical trials within the last 3 months before the beginning of this study were excluded. In addition, men were excluded if they had any medications, had a poor understanding of daily intervention, and had a history of serious disease including cancerous disease.

**Figure 1:**
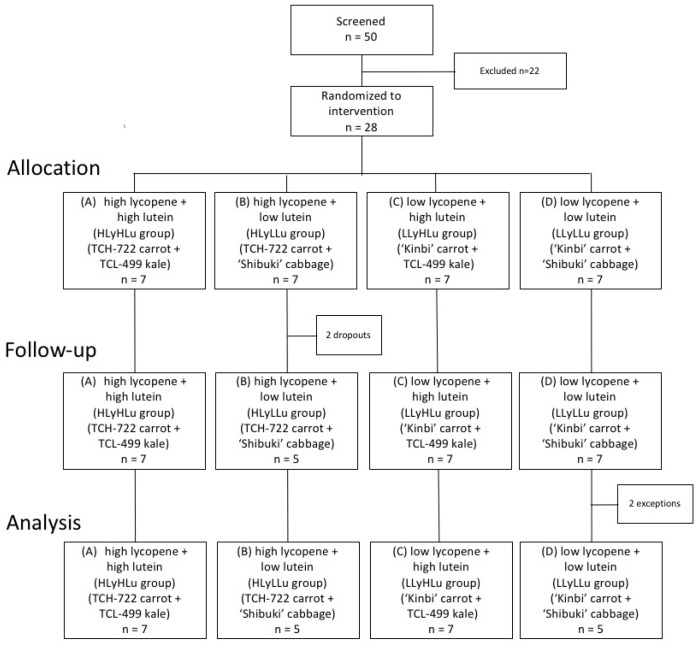
Enrollment and follow-up of obese men who participated in the present randomized, double-blind study.

After successful screening, 28 subjects out of 50 candidates were enrolled in this study. Eligible subjects were randomly assigned and equally distributed to receive one of four interventions: (A) TCH-722 carrot and TCL-499 kale pastes (high lycopene + high lutein or HLyHLu group), (B) TCH-722 carrot and “Shibuki” cabbage pastes (high lycopene + low lutein or HLyLLu group), (C) “Kinbi” carrot and TCL-499 kale pastes (low lycopene + high lutein or LLyHLu group), or (D) “Kinbi” carrot and cabbage pastes (low lycopene + low lutein or LLyLLu group). During the 8-week intervention phase, each subject received once daily, before breakfast, an identical beverage containing 400 g of the testing pastes (Figure 1). The proper intake of the beverage was monitored by collecting the self-recorded paper of beverage intake every week during intervention periods. The experimental conditions were registered with the University Hospital Medical Information Network (UMIN) Center (www.umin.ac.jp) and the UMIN Clinical Trials Registry (UMIN-CTR) Identifier is UMIN000014482.

### 2.2. Physical Assessment and Biochemical Analysis

All enrolled subjects completed a brief physical examination including the measurement of waist circumference according to the International Society for Advancement of Kinanthropometry (ISAK) guidelines. To evaluate the visceral adipose tissue (VAT), we measured the visceral fat level using the Body Composition Analyzer, INNER SCAN50 BC-320 (Tanita Co., Tokyo, Japan), and applied the bioelectrical impedance analysis (BIA) method which is in good correlation with the assessment of visceral fat area using a computed tomography (CT) scan at the umbilical level [30].

Serum triglycerides, total cholesterol, HDL cholesterol, LDL cholesterol, and plasma fasting glucose levels were measured at baseline and post-intervention by routine clinical chemistry. In addition, as an index of the antioxidant status, oxidative stress, and systemic inflammation, soluble circulating lectin-like oxidized low-density lipoprotein receptor-1 (sLOX-1), coenzyme Q10 (CoQ10), interleukin (IL)-6, and tumor necrosis factor (TNF)-α were also evaluated at baseline and post-intervention, measured by BioMarker Science Co., Ltd. (Kyoto, Japan). In brief, the serum levels of IL-6 and TNF-α, and the plasma level of sLOX-1 were measured using an enzyme-linked immunosorbent assay (ELISA), whereas the reduced form of coenzyme Q10 (ubiquinol) and the oxidized form of CoQ10 (ubiquinone) were measured in the serum samples according to previously described methods [31]. Regarding CoQ10, its value was expressed as the percentage of the oxidized form of CoQ10 (ubiquinone) in the total CoQ10 (% CoQ10).

### 2.3. Chemical Reagents

α-carotene and β-carotene standards were purchased from Wako Pure Chemical Industries, Ltd. (Osaka, Japan). Lutein standard was purchased from CaroteNature GmbH (Ostermundigen, Switzerland). Lycopene standard was purchased from Sigma-Aldrich Co. LLC (St. Louis, MO, USA). Special grade n-Hexane, acetone, ethanol, toluene, and pyrogallol were purchased from Wako Pure Chemical Industries, Ltd. HPLC grade methyl tert-butyl ether, acetonitrile, and methanol were purchased from Wako Pure Chemical Industries, Ltd. Cellulose was purchased from Amano Enzyme Inc. (Nagoya, Japan). Pectinase was purchased from Yakult Pharmaceutical Industry Co., Ltd. (Tokyo, Japan). Citric acid was purchased from Showa Kako Co., Ltd., (Osaka, Japan). Pectin was purchased from Unitec Foods Co., Ltd., (Tokyo, Japan).

### 2.4. Plant Materials

**Figure 2:**
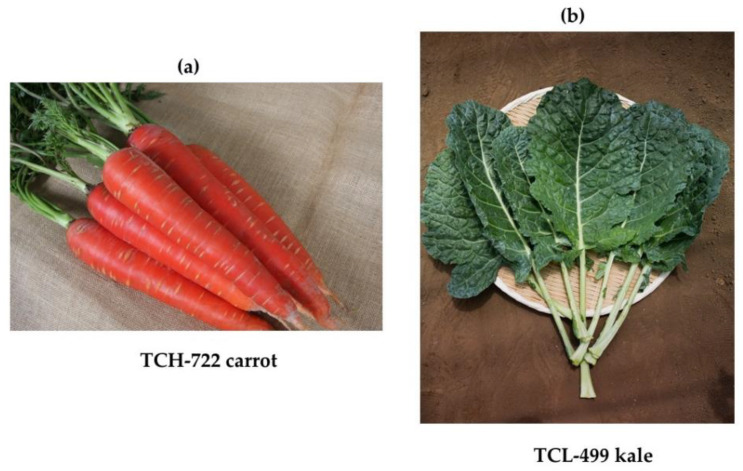
Representative pictures of TCH-722 carrots (**a**) and TCL-499 kale (**b**).

In this study, we used a carrot cultivar, highly enriched in lycopene (Figure 2A), and a kale cultivar highly enriched in lutein (Figure 2B). Carrots in general do not contain lycopene; however, for this study, we chose a carrot cultivar containing high level of lycopene in addition to α-carotene, β-carotene, and lutein. We also included a kale cultivar containing a high level of lutein in addition to β-carotene so as to evaluate the effects of various carotenoids in human health.

The carrot (*Daucus carota subsp. Sativus*) cultivar used in our experiments was “TCH-722” (Takii Co., Ltd. Kyoto, Japan), and the kale (*Brassica oleracea var. acephala*) cultivar used was “TCL-499” (Takii Co., Ltd.). As placebo vegetables, we used carrot “Kinbi” (Mikado Kyowa seed Co., Ltd., Chiba, Japan) for the carrot TCH-722, and cabbage (*Brassica oleracea var. capitata*) “Shibuki” (Ishii Seed Growers Co., Ltd. Shizuoka, Japan) for the kale TCL-499. Harvested vegetables were sterilized with superheated steam at 120 °C for 30 min and smashed and minced to paste. Pastes were frozen and stored at −20 °C until processed to beverages for the clinical trial.

### 2.5. Preparation of Beverages

We produced four types of beverages, each including two paste types. The two-paste combinations were as follows: (A) TCH-722 carrot and TCL-499 kale pastes (HLyHLu group), (B) TCH-722 carrot and “Shibuki” cabbage pastes (HLyLLu group), (C) “Kinbi” carrot and TCL-499 kale pastes (LLyHLu group), and (D) “Kinbi” carrot and cabbage pastes (LLyLLu group).

Carrot paste (400 kg) and kale or cabbage paste (200 kg) were mixed and heated to 35 °C. Cellulose (3 kg) and pectinase (3 kg) enzyme were added to the paste-mixtures and incubated at 45 °C for 60 min. Next, the paste-mixtures were sterilized at 70 °C for 60 min. Boiled water (400 L), pectin (8 kg), and citric acid (3.25 kg) were added to the paste mixture. Citric acid was used to adjust the pH (<4). Two hundred milliliters of paste mixtures were bottled in aluminum-metallized pouches and sterilized at 90 °C for 40 min. Beverages were stored at 20–25 °C.

### 2.6. Analysis of Dietary Fiber and Carotenoids in Beverages

The dietary fiber content of the beverages was determined using the AOAC Official Method of Analysis 985.29 [32]. Samples of the vegetables were minced using a domestic food processor. Water (5 mL) and pyrogallol (2 g) were added to the minced samples (1 g) and homogenized by polytron homogenizer (PT-3000; Kinematica Switzerland). Samples of the beverages (2.5 g) were mixed with pyrogallol (2 g) and water (1 mL). Forty milliliter of n-Hexane/Acetone/Ethanol/Toluene (10:7:6:7) and 20 mL of ethanol were added to the vegetable or beverage samples, extracted with supersonic wave for 10 min, and filled up to 100 mL with ethanol. The carotenoid extract was filtered using a 0.2 μm filter (Advantec Toyo Kaisha, Ltd. Tokyo, Japan) and analyzed by HPLC. Finally, the carotenoid content of vegetables and beverages for clinical test was analyzed by HPLC.

### 2.7. HPLC Apparatus and Conditions

Lutein, α-carotene, β-carotene, and lycopene in the beverages were quantified using an HPLC system consisting of an L-2130 pump, an L-2300 column oven, an L-2400 UV-VIS detector, an L-2200 auto-sampler (Hitachi High-Technologies corporation, Tokyo, Japan), and a personal computer equipped with EZChrome Elite Chromatography Data system software (Scientific Software Inc., Pleasanton, CA, USA). All the components were detected using a YMC carotenoid column (4.6 × 250 mm; YMC Co., Ltd. Kyoto, Japan). A mixture of methyl tert-butyl ether/acetonitrile (85:15, vol/vol) was used as mobile phase A, and methanol was used as mobile phase B. The applied gradient elution was as follows: mobile phase A started at 10% at a flow rate of 1.5 mL/min, increased linearly to 100% A from 0 min to 9.0 min and kept for 3.0 min, and finally changed to 10% A from 12 min to 12.1 min and kept for 3.9 min. The column temperature was set at 30 °C. The injection volume was 20 μL. All components were detected at 475 nm using an L-2400 UV-VIS detector. Their contents were quantified from their peak areas using the standard curves. Confirmation of carotenoid identities was done by comparison of the retention time with the obtained carotenoid standards. Typical retention times for the standards is as follows, lutein: 5.01 min, α-carotene: 7.71 min, β-carotene: 8.12 min, lycopene: 11.14 min. These analyses were performed in duplicate.

### 2.8. DNA Microarray Experiments and Data Analysis

Total RNA was isolated from whole blood using the PAXgene Blood RNA Kit (QIAGEN-PreAnalytix GmbH, Hombrechtikon, Switzerland). The quality and quantity of purified total RNA were confirmed by agarose gel electrophoresis and spectrophotometry, respectively. One sample from LLyHLu group was not used for DNA microarray analysis due to its markedly low integrity of total RNA. Biotinylated cRNA was obtained from 200 ng of purified total RNA using GeneChip^®^ 3′ IVT PLUS reagent kit (Affymetrix, Santa Clara, CA, USA). The cRNA was subsequently fragmented and hybridized to a GeneChip^®^ Human Genome U133 Plus 2.0 Array (Affymetrix). The arrays were washed and labeled with streptavidin-phycoerythrin using the GeneChip^®^ Hybridization, Wash, and Stain Kit and the Fluidics Station 450 system (Affymetrix). Fluorescence was detected using a GeneChip^®^ Scanner 3000 7G (Affymetrix). All experimental procedures were carried out according to the manufacturer’s instructions. All microarray data are Minimum Information About a Microarray Experiment (MIAME) compliant and have been deposited in a MIAME compliant database, the National Center for Biotechnology Information (NCBI) Gene Expression Omnibus (http://www.ncbi.nlm.nih.gov/geo/, GEO Series accession number GSE151683), as detailed on the FGED Society website (http://fged.org/projects/miame/).

The Affymetrix GeneChip^®^ Command Console (AGCC) software was used to reduce the array images to the intensity of each probe (CEL files). CEL files were quantified using the Factor Analysis for Robust Microarray Summarization (FARMS) algorithm [33] with the statistical language R [34] and Bioconductor [35]. Principal component analysis [36] was performed using the prcomp() function and ggplot2 package [37] in R.

### 2.9. Statistical Analysis

All values are expressed as mean ± standard deviation (SD). Physical data, blood carotenoid, and blood oxidative markers were analyzed by paired t-test. *p* values less than 0.05 were considered statistically significant. Statistical analyses of the experimental data were performed with GraphPad Prism 8 (GraphPad Software Inc., La Jolla, CA, USA) for Macintosh.

## 3. Results

### 3.1. Clinical Characteristics

Twenty-eight subjects were randomly assigned to four groups and consumed the test beverage for 8 weeks. After randomization, 2 men of group B (HLyLLu group) withdrew from the study, one because of hospitalization after a traffic injury and the other because of residence relocation. From group D (LLyLLu group), 2 men were also excluded from the analysis, one because the received serum sample was inadequate and the other because of pharmacological treatment commencement for a non-related with this study issue. Thus, a total of 24 participants whose daily intake of a test beverage could be confirmed completed this study without any adverse events (Figure 1). The average age of the subjects in each of the four groups was as follows: (A) HLyHLu group: 49.0 ± 6.2; (B) HLyLLu group: 46.8 ± 8.8; (C) LLyHLu group: 44.9 ± 5.2; (D) LLyLLu group: 45.8 ± 9.7, and there were no significant differences among all groups.

### 3.2. Carotenoids and Dietary Fiber of Beverages

All subjects received once daily a test beverage consisting of 400 g of pastes. We analyzed the contents of carotenoids and the dietary fibers contained in test beverage (Table 1). The HLyHLu group (A) and HLyLLu group (B) beverages containing TCH-722 carrots had a higher lycopene content than the other group beverages because the TCH-722 carrots contained lycopene in addition to α-/β-carotene. The HLyHLu group (A) and LLyHLu group (C), including kale, also contained higher levels of lutein and dietary fibers compared with the other groups.

### 3.3. Evaluation of Plasma Carotenoids Level

We measured the plasma levels of carotenoids to evaluate their absorption after intake of the test beverages (Table 2). In HLyHLu group (A), the plasma levels of all carotenoids were markedly increased (*p* < 0.01). The level of α-carotene was significantly increased in both HLyHLu group (A) and HLyLLu group (B) which included TCH-722 carrots; β-carotene was also increased in all groups although its quantity fluctuated. The plasma level of lycopene showed no significant increase except for the HLyHLu group (A), though the plasma level of lycopene was likely to increase in the HLyLLu group (B) which included the TCH-722 carrots. In addition, the plasma level of lutein showed no significant increased except for the HLyHLu group (A), though these levels were likely to increase in the LLyHLu group (C) which included the TCL-499 kale. The small number of subjects may be responsible for the absence of significant increase in lycopene in the HLyLLu group (B) and lutein in the LLyHLu group (C).

### 3.4. Physical Assessments

We measured the body weight, waist circumference, body mass index, and visceral fat indicator at baseline and after 8 weeks of intervention as shown in Table 3. Although the LLyLLu group (D) seemed to be likely obese, these parameters had no significant differences at baseline among all groups. Waist circumference was reduced after the consumption of the test beverage in HLyLLu group (B) (*p* = 0.02). More importantly, the visceral fat level was significantly decreased in all groups after 8 weeks intervention. Additionally, we could find no association between the increase of each carotenoid and the decrease of visceral fat level (data not shown). There was no significant variation in body weight and body mass index in this study. In addition, there were no obvious changes regarding the blood pressure and cardiac rate during this investigation (data not shown).

### 3.5. Biochemical Analysis of Blood

We performed several biochemical tests on the blood as shown in Table 4. At the level of fasting glucose, triglycerides, total cholesterol, and HDL cholesterol, there were no obvious differences or alternations at baseline and after 8 weeks of intervention among all groups. The LDL cholesterol level was significantly increased after 8 weeks of intervention only in the LLyHLu group (C), although its underlying mechanism is unclear.

We also measured blood oxidative markers, shown in Table 5. Although the plasma level of sLOX-1 did not differ significantly among all groups, the % CoQ10 was significantly reduced after 8 weeks intervention in all groups. In addition, we also measured the serum levels of TNF-α and IL-6, as the proinflammatory cytokines associated with the adipose tissue [38]. In the present study, the production of these cytokines showed no obvious differences or alternation at baseline and after 8 weeks intervention among all groups.

### 3.6. Changes in Gene Expression Profiles in Whole Blood

**Figure 3:**
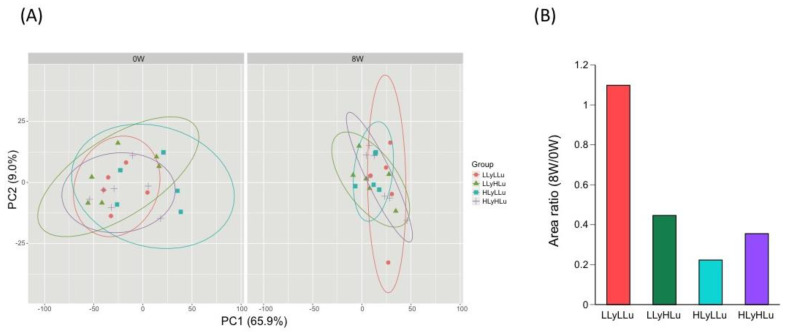
Comparison of the gene expression profiles in the subjects’ blood before (0 W) and after (8 W) the test. (**A**) Principal component analysis was performed using DNA microarray data from individual blood. (**A**) before the test (0 W); (**B**) after the test (8 W). The proportion of variance is indicated in parenthesis after each principal component. The ellipses represent 90% confidence intervals of the group. (**B**) The area ratio of 8W to 0W probability ellipses for each group.

Gene expression profiles in whole blood before and after ingestion of vegetable drinking were evaluated by principal component analysis. Before the test (0 W), sample plots in each group dispersed uniformly in PC1-PC2 plot (Figure 3A, left panel). However, after 8 weeks ingestion of beverages (8 W), gene expression profiles in blood became close to each other except for those in LLyLLu group (D) (Figure 3B, right panel). The area of the probability ellipses in the Figure 3A for each group were expressed as a ratio of 8W to 0W (Figure 3B). These results suggest that the carotenoids in the drink affect the gene expression profile in subjects’ blood.

## 4. Discussion

In the present study, we performed an intervention pilot study on the effects of short-term intake of beverages containing carotenoids produced from vegetables, in obese middle-aged Japanese men. The most striking feature of this investigation is the significant decrease of visceral fat level by the intake of beverages that contained carotenoids. In addition, the 8 week-ingestion of carotenoids inhibited the oxidative stress status, as this was revealed by the inhibition of % CoQ10. These results may be indicative of the usefulness of vegetables with high carotenoid-content and their functionality on the health of individuals that have a high risk for developing metabolic syndrome, although this study only considered obese middle-aged Japanese men. On the contrary, the inhibition of visceral fat level and oxidative stress status was observed in low lycopene + low lutein (LLyLLu) group as well as in other groups receiving the test beverages derived from carotenoid-rich vegetables. We believe these results may be obtained by the promotion of behavior modification to healthy lifestyle through the participation in this clinical investigation, although these findings should be investigated in detail in future. In addition, these findings may be influenced by the load of dietary fiber in all groups. Nevertheless, it was considered to represent a difficult aspect of the clinical trial using vegetables as the test samples.

Metabolic syndrome is one of the risk factors associated with lifestyle diseases, and it is also known to be related to diet. In particular, the intake of carotenoids has been reported to be negatively correlated with the development of metabolic syndrome and lifestyle-related diseases [39]. Many vegetables contain carotenoids, and humans consume carotenoids through vegetable intake in their daily diet. Therefore, it is necessary to elucidate the effects of carotenoid-containing vegetables on the body and the metabolic syndrome. However, the effects of carotenoid-containing vegetables on the metabolic syndrome in the Japanese population remain unclear. To analyze the impact of multiple vegetable-contained carotenoids, especially that of lycopene and lutein, on the metabolic syndrome, we designed an RCT trial with two combinations of four different vegetables in four groups. To evaluate the health effects of carotenoid-rich vegetables, we tested beverages that contained whole vegetables. In our results, no obvious adverse events were observed reflecting thus, the safety of the vegetables used.

For the evaluation of the visceral adipose tissue, a CT scan at the umbilical level is usually performed in order to assess the visceral fat area. However, the use of CT scan is no cost-effective, and it also includes the need of radiation exposure. In contrast, the bioelectrical impedance analysis (BIA) method used in the present study is a simple and noninvasive procedure for the assessment of the visceral fat accumulation, and an excellent correlation has been observed in the estimation of visceral fat accumulation between abdominal BIA method and CT scan [30]. Our results showed that the visceral fat level was significantly decreased in all groups. However, the waist circumference was reduced in only the HLyLLu (high lycopene + low lutein) group, suggesting that carotenoids alone may not have contributed in the reduction of visceral fat and waist circumference. Vegetables contain various secondary metabolites with anti-metabolic effects, such as polyphenols and glucosinolates, in addition to carotenoids; thus, it is possible that these substances may have contributed to the reduction of the waist visceral fat and circumference, in combination with the carotenoids.

In this study, we could not confirm a significant improvement of serum triglycerides, total cholesterol, HDL cholesterol, LDL cholesterol, and plasma fasting glucose, which are important components for the diagnosis of the metabolic syndrome. Previous studies reported that sLOX-1 was related to adipocyte metabolism, inflammation, and immune response associated with obesity [40,41]; therefore, we tested the plasma level of sLOX-1. However, there was no obvious alteration after the 8 week-intervention in all four groups. In contrast, a decrease in the % CoQ10 level was observed in all groups. This may indicate that the reduction of oxidative stress due to the ingestion of carotenoids appears relatively earlier than the improvement of the metabolic syndrome serum-markers. The redox balance of CoQ10 in the human serum is a good marker of oxidative stress because the reduced form of CoQ10 (ubiquinol) is very sensitive to oxidation and is quantitatively converted to its oxidized form (ubiquinone) [42]. Actually, elevation of the % CoQ10 was confirmed in patients with various diseases, including sepsis, hepatitis, cirrhosis, hepatoma, Parkinson’s disease, juvenile fibromyalgia, amyotrophic lateral sclerosis (ALS), and post-cardiac arrest syndrome [43]. Although further studies on the relationship between vegetable intake and the inhibition of CoQ10 oxidation are required, the elevation of plasma concentration of carotenoids may contribute to the improvement of the redox status.

Furthermore, it has been demonstrated that the adipose tissue systemically releases proinflammatory cytokines such as TNF-α and IL-6, which activate the macrophages in the adipose tissue of obese individuals [44]. These proinflammatory cytokines not only impair the action of insulin in metabolic tissues, but also favor cancer development [38]. However, even though we also checked for these cytokines, their production did not show any obvious alteration after the 8 week-intervention period, in all groups. In contrast, principal component analysis of the gene expression profiles in whole blood, before and after ingestion of carotenoids, brought them close to each other except for those from the LLyLLu (low lycopene + low lutein) group, indicating that the ingestion of carotenoids could indeed affect the gene expression profile.

This study has several limitations: The clinical investigation included only a limited number of enrolled subjects, the interventional period was short (8 weeks), and it was performed in a single center, the reason being that it served as an exploratory study prior to a large clinical trial. In particular, because we could not estimate the sample size ahead of time, it seems that the small sample size may have affected the results and had not enough power to produce the outcome. In addition, dietary intake as a confounding factor could not be adjusted since the survey of dietary intake has not been conducted in this trial. However, it was revealed in the present study that the consumption of vegetables containing high carotenoids increased blood carotenoids. These findings warrant the need for further evaluation of the usefulness of vegetables rich in carotenoids in patients with metabolic syndrome, including a large number of subjects, in the near future.

## 5. Conclusions

In summary, this study revealed that in middle-aged men with a BMI of 25 or higher, the consumption of carotenoid-rich vegetables led to increased serum levels of carotenoids and reduced levels of visceral adiposity and oxidative stress. Furthermore, the consumption of carotenoid-containing vegetables significantly affected the gene expression profile in the blood. These results may indicate the beneficial effects of vegetable intake, including carotenoids intake, for the prevention of the metabolic syndrome in the Japanese population. To clarify the mechanisms by which the consumption of carotenoid-rich vegetables can lead to these findings, a larger-scale clinical trial is warranted in the near future.

## Figures and Tables

**Table 1:** The contents of carotenoids and dietary fibers contained in the test beverages produced from vegetables.

	(A) High Lycopene + High Lutein (HLyHLu Group) (TCH-722 Carrot + TCL-499 Kale)	(B) High Lycopene + Low Lutein (HLyLLu Group) (TCH-722 Carrot + ‘Shibuki’ Cabbage)	(C) Low Lycopene + High Lutein (LLyHLu Group) (‘Kinbi’ Carrot + TCL-499 Kale)	(D) Low Lycopene + Low Lutein (LLyLLu Group) (‘Kinbi’ Carrot + ‘Shibuki’ Cabbage)
α-carotene (mg/day)	1.88	1.8	0.12	0.12
β-carotene (mg/day)	13.44	10.92	6.92	3.72
Lycopen (mg/day)	7.56	8.6	0	0
Lutein (mg/day)	1.96	0.12	2.48	0.16
Fiber (g/day)	6.4	4.4	6.8	4

**Table 2:** The assessment of plasma carotenoids levels at baseline and after 8 weeks of intervention.

	(A) High Lycopene + High Lutein (HLyHLu Group) (TCH-722 Carrot + TCL-499 Kale)		(B) High Lycopene + Low Lutein (HLyLLu Group) (TCH-722 Carrot + ‘Shibuki’ Cabbage)		(C) Low Lycopene + High Lutein (LLyHLu Group) (‘Kinbi’ Carrot + TCL-499 Kale)		(D) Low Lycopene + Low Lutein (LLyLLu Group) (‘Kinbi’ Carrot + ‘Shibuki’ Cabbage)	
	0 wks	8 wks	0 wks	8 wks	0 wks	8 wks	0 wks	8 wks
α-carotene (μg/dL)	5.0 ± 4.93	14.1 ± 5.42 **	5.2 ± 5.44	14.1 ± 5.51 *	7.1 ± 8.17	8.1 ± 7.28	5.12 ± 5.11	4.02 ± 3.19
β-carotene (μg/dL)	15.6 ± 14.34	52.5 ± 38.37 **	10.7 ± 7.99	30.3 ± 13.53 **	21.0 ± 14.50	52.0 ± 32.52 **	16.4 ± 6.90	26.5 ± 11.02 *
Lycopen (μg/dL)	45.0 ± 28.90	74.1 ± 27.28 **	35.0 ± 26.42	54.7 ± 28.40	38.4 ± 28.05	47.6 ± 46.21	35.5 ± 28.56	39.52 ± 42.57
Lutein (μg/dL)	39.6 ± 11.10	66.5 ± 22.64 **	33.7 ± 10.34	44.1 ± 19.32	37.9 ± 7.82	69.3 ± 27.34	41.3 ± 28.05	38.1 ± 26.19

All values represent the means (± standard deviation, SD). * *p* < 0.05, ** *p* < 0.01 vs. values at baseline.

**Table 3:** The physical assessment at baseline and after 8 weeks of intervention.

	(A) High Lycopene + High Lutein (HLyHLu Group) (TCH-722 Carrot + TCL-499 Kale)		(B) High Lycopene + Low Lutein (HLyLLu Group) (TCH-722 Carrot + ‘Shibuki’ Cabbage)		(C) Low Lycopene + High Lutein (LLyHLu Group) (‘Kinbi’ Carrot + TCL-499 Kale)		(D) Low Lycopene + Low Lutein (LLyLLu Group) (‘Kinbi’ Carrot + ‘Shibuki’ Cabbage)	
	0 wks	8 wks	0 wks	8 wks	0 wks	8 wks	0 wks	8 wks
Body Weight (kg)	81.3 ± 10.48	81.5 ± 10.16	80.6 ± 4.08	80.4 ± 3.98	86.5 ± 13.49	86.7 ± 13.70	95.8 ± 18.63	96.6 ± 18.54
Waist Circumference (cm)	96.9 ± 8.23	96.7 ± 9.41	98.0 ± 5.00	95.8 ± 4.66 *	97.4 ± 7.32	97.6 ± 9.08	104.4 ± 14.94	105.4 ± 16.67
Body Mass Index (BMI)	28.1 ± 3.09	28.2 ± 3.07	27.9 ± 1.78	27.8 ± 1.72	28.2 ± 3.05	28.2 ± 3.16	31.4 ± 5.42	31.6 ± 5.45
Visceral Fat Level	15.2 ± 1.89	14.2 ± 2.25 **	14.8 ± 1.52	14.0 ± 1.27 **	14.6 ± 2.59	13.8 ± 2.91 *	17.0 ± 2.72	16.4 ± 2.75 **

All values represent the means (± standard deviation, SD). * *p* < 0.05, ** *p* < 0.01 vs. values at baseline.

**Table 4:** The assessment of fasting glucose, HDL/LDL/total cholesterol, and triglycerides at baseline and after 8 weeks of intervention.

	(A) High Lycopene + High Lutein (HLyHLu Group) (TCH-722 Carrot + TCL-499 Kale)		(B) High Lycopene + Low Lutein (HLyLLu Group) (TCH-722 Carrot + ‘Shibuki’ Cabbage)		(C) Low Lycopene + High Lutein (LLyHLu Group) (‘Kinbi’ Carrot + TCL-499 Kale)		(D) Low Lycopene + Low Lutein (LLyLLu Group) (‘Kinbi’ Carrot + ‘Shibuki’ Cabbage)	
	0 wks	8 wks	0 wks	8 wks	0 wks	8 wks	0 wks	8 wks
Fasting glucose (mg/dL)	94.0 ± 11.42	89.3 ± 7.70	92.0 ± 11.40	91.0 ± 9.38	93.4 ± 6.00	92.7 ± 5.65	88.2 ± 9.36	94.8 ± 9.23
HDL cholesterol (mg/dL)	46.1 ± 6.41	45.7 ± 8.62	59.4 ± 30.29	53.8 ± 22.59	46.9 ± 5.84	48.4 ± 6.37	41.8 ± 6.83	39.4 ± 9.15
LDL cholesterol (mg/dL)	146.1 ± 30.47	153.9 ± 30.32	121.0 ± 39.56	130.4 ± 28.05	136.0 ± 34.73	149.7 ± 38.25 *	158.0 ± 20.16	153.2 ± 46.80
Total cholesterol (mg/dL)	219.3 ± 31.98	225.1 ± 27.22	210.2 ± 13.61	211.4 ± 11.06	205.6 ± 38.41	213.9 ± 42.53	246.6 ± 35.24	230.6 ± 42.0
Triglycerides (mg/dL)	165.1 ± 66.87	203.1 ± 71.30	149.2 ± 112.92	180.4 ± 112.53	111.1 ± 42.53	135.1 ± 30.46	212.8 ± 105.55	323.8 ± 261.61

All values represent the means (± standard deviation, SD). * *p* < 0.05 vs. values at baseline.

**Table 5:** The assessment of oxidative markers and proinflammatory cytokines (IL-6 and TNF-α) at baseline and after 8 weeks of intervention.

	(A) High Lycopene + High Lutein (HLyHLu Group) (TCH-722 Carrot + TCL-499 Kale)		(B) High Lycopene + Low Lutein (HLyLLu Group) (TCH-722 Carrot + ‘Shibuki’ Cabbage)		(C) Low Lycopene + High Lutein (LLyHLu Group) (‘Kinbi’ Carrot + TCL-499 Kale)		(D) Low Lycopene + Low Lutein (LLyLLu Group) (‘Kinbi’ Carrot + ‘Shibuki’ Cabbage)	
	0 wks	8 wks	0 wks	8 wks	0 wks	8 wks	0 wks	8 wks
sLOX-1 (ng/L)	525.9 ±185.61	606.9 ± 272.91	525.9 ± 212.21	439.3 ± 143.26	465.6 ± 245.41	391.3 ± 160.98	643.3 ± 263.59	684.7 ± 283.55
%CoQ10 (%)	10.7 ± 1.47	8.0 ± 1.73 *	9.4 ± 1.38	7.2 ± 2.09 *	10.6 ± 1.17	7.5 ± 1.34 **	13.1 ± 4.96	8.9 ± 2.70 *
IL-6 (pg/mL)	1.3 ± 1.1	1.1 ±0.62	1.2 ± 1.09	1.3 ± 1.32	1.1 ± 1.35	0.9 ± 1.09	1.0 ± 0.71	1.2 ± 0.6
TNF-α (pg/mL)	1.4 ± 0.47	1.3 ± 0.47	1.2 ± 0.12	1.0 ± 0.20	1.4 ± 0.38	1.1 ± 0.23	1.5 ± 0.48	1.3 ± 0.43

All values represent the means (± standard deviation, SD). * *p* < 0.05, ** *p* < 0.01 vs. values at baseline.
